# A highly sensitive quantitative real-time PCR assay based on the *groEL* gene of contemporary Thai strains of *Orientia tsutsugamushi*

**DOI:** 10.1111/j.1469-0691.2008.02671.x

**Published:** 2009-05

**Authors:** D H Paris, N Aukkanit, K Jenjaroen, S D Blacksell, N P J Day

**Affiliations:** 1 Faculty of Tropical Medicine, Mahidol UniversityBangkok, Thailand; 2 Centre for Clinical Vaccinology and Tropical Medicine, Nuffield Department of Clinical Medicine, Churchill Hospital, University of OxfordOxford, UK

**Keywords:** Diagnostics, *groEL*, heat shock protein, phylogenetics, real-time PCR, scrub typhus

## Abstract

Partial nucleotide sequences (459 bp) of the *groEL* gene (encoding the 60-kDa heat shock protein, HSP60) from 23 contemporary isolates of *Orientia tsutsugamushi* isolated from patients with acute scrub typhus in Thailand were compared with 16 reference strain sequences to evaluate the potential of *groEL* as a conserved and representative target for molecular diagnostics.. Overall nucleotide identity within all available *O. tsutsugamushi* isolates (*n* = 39) was 98.8% (range: 95.0–100), reflecting a high degree of conservation; nucleotide identities were 67.5% and 65.6%, respectively, when typhus and spotted fever group rickettsiae were included.. A highly sensitive and quantitative real-time PCR assay was designed and evaluated using 61 samples, including buffy coats from patients in Thailand and Laos. Reliable and accurate quantitation of bacterial loads allows further investigation of other diagnostic methods and may lead to an improved understanding of the pathophysiology of acute scrub typhus, an important but under-recognized disease.

## Introduction

The *Rickettsiaceae* family consists of a group of highly fastidious, obligate intracellular Gram-negative organisms. They are divided into three groups, based on antigenic reactivity—the scrub typhus group, typhus group (TG) and spotted fever group (SFG). Scrub typhus, caused by *Orientia tsutsugamushi*, and murine typhus, caused by *Rickettsia typhi*, are the most common forms of Typhus in rural Thailand and Laos, accounting for 20–30% of undifferentiated fevers [1–4].

Problems in under-recognition of rickettsial illnesses, mainly due to diagnostic difficulties, lead to delay and errors in patient management. The development of rapid, inexpensive and accurate diagnostic methods is necessary, both to improve diagnosis and to promote awareness of these potentially serious but treatable diseases in highly populous rural areas of Southeast Asia.

With the increasing availability of gene sequences, allowing the exploitation of more gene-based targets, molecular assays have been developed and evaluated for the diagnosis of acute scrub typhus. A common target gene used in nested conventional as well as real-time PCR assays, encodes the 56-kDa outer membrane protein [5,6]. Another target gene encodes the 47-kDa outer membrane protein, used in a real-time PCR assay [7].

Quoted sensitivities for the nested 56-kDa assays range from 62% to 90%, with specificities approaching 100% when compared to the reference standard immunofluorescence assay (IFA) [5,6,8,9]. Recent studies have characterized the 60-kDa heat shock protein GroEL of α-proteobacteriaceae as a molecular indicator of various forms of cellular stress. GroEL production is upregulated during the early period of infection, leading to high-level expression of essential proteins in eubacterial genomes and in eukaryotic organelles [10,11]. The most prominent protein of *Rickettsia conorii* (SFG), revealed by two-dimensional PAGE proteomic analysis and reacting with antibodies in rabbit and patient sera, was a 60-kDa protein identified as GroEL [12].

The corresponding gene has been proposed as a target for molecular diagnostics for differentiation between members of the genus *Rickettsia* [13] and the family *Anaplasmataceae* [14]. Recently, Park *et al.* described the use of a conventional duplex PCR assay, based on the *groEL* gene, for the detection of rickettsiae and the identification of *O. tsutsugamushi* [15]. This assay was evaluated using a limited number of *O. tsutsugamushi* reference type strains, including strains Karp, Kato, Kawasaki, Gilliam and Boryong.

The *groEL* nucleotide sequences of 23 contemporary *in vitro* isolates of *O. tsutsugamushi* isolated from patients with scrub typhus in Thailand were determined and the corresponding amino acid sequences deduced. The sequences were compared with those of reference strains to evaluate the potential of *groEL* as a conserved and representative molecular target. Using these data, together with previously published nucleotide sequence information, a novel and highly sensitive real-time PCR assay was developed for the detection and quantitation of *O. tsutsugamushi*.

## Materials and Methods

### Clinical samples

The *O. tsutsugamushi* isolates cultured in this study (Table 1) were collected from scrub typhus patients (in 5 mL of full blood containing EDTA) at two sites in Thailand during 2004–2005. Previous antibiotic use was an exclusion criterion for sample collection. The isolates were cultivated in VERO cell monolayers in 25-cm^2^ polystyrene tissue culture flasks (Becton Dickinson, Franklin Lakes, NJ, USA) containing RPMI-1640 medium supplemented with l-glutamine, HEPES (2 mM) and fetal bovine serum (FCS Gold; PAA, Laboratories GmbH, Pasching, Austria) (10%, v/v)). Cultures of *O. tsutsugamushi* were incubated at 35°C in a 5% CO_2_ atmosphere [16]. When the cytopathic plaque-formation reached 90–100% confluency of the whole monolayer, the cells were harvested, pelleted and stored at −80°C.

**TABLE 1:** Details of isolates and strains used in this study

Available *groEL* gene sequences (HSP 60 kDa)								

New Thailand isolates						NCBI strains		
	
Species	Isolate	NCBI accession number	Strain^a^	Country	Year of isolation	Species	Strain	NCBI accession number
*Orientia tsutsugamushi*	UT76	EF551292	Karp	North-eastern Thailand	2003	*O. tsutsugamushi*	Boryong	AY059015
*O. tsutsugamushi*	UT125	EF551293	Gilliam	North-eastern Thailand	2003	*O. tsutsugamushi*	Karp	M31887
*O. tsutsugamushi*	UT144	EF551294	Gilliam	North-eastern Thailand	2004	*O. tsutsugamushi*	Gilliam	AY191585
*O. tsutsugamushi*	UT150	EF551295	Karp	North-eastern Thailand	2004	*O. tsutsugamushi*	Hwasung	AY191589
*O. tsutsugamushi*	UT167	EF551296	Karp	North-eastern Thailand	2004	*O. tsutsugamushi*	Kato	AY191586
*O. tsutsugamushi*	UT169	EF551297	Karp	North-eastern Thailand	2004	*O. tsutsugamushi*	Kawasaki	AY191587
*O. tsutsugamushi*	UT176	EF551298	Karp	North-eastern Thailand	2004	*O. tsutsugamushi*	Youngworl	AY191588
*O. tsutsugamushi*	UT177	EF551299	Karp	North-eastern Thailand	2004	*Rickettsia typhi*	Wilmington	AY191590
*O. tsutsugamushi*	UT196	EF551300	Gilliam	North-eastern Thailand	2004	*R. prowazekii*	Breinl	Y15783
*O. tsutsugamushi*	UT213	EF551301	Karp	North-eastern Thailand	2004	*R. akari*	ATCC VR-148	AY059013
*O. tsutsugamushi*	UT219	EF551302	Karp	North-eastern Thailand	2004	*R. belli*	RML369-C	NC 007 940
*O. tsutsugamushi*	UT221	EF551303	Karp	North-eastern Thailand	2004	*R. conorii*	Malish	AY059012
*O. tsutsugamushi*	UT302	EF551304	Karp	North-eastern Thailand	2004	*R. helvetica*	NS	DQ442911
*O. tsutsugamushi*	UT329	EF551305	Gilliam	North-eastern Thailand	2004	*R. japonica*	ATCC VR-1363	AF432181
*O. tsutsugamushi*	UT332	EF551306	Karp	North-eastern Thailand	2004	*R. rickettsii*	Bitterroot	U96733
*O. tsutsugamushi*	UT336	EF551307	Karp	North-eastern Thailand	2004	*R. sibirica*	ATCC VR-151	AY059014
*O. tsutsugamushi*	UT340	EF551308	Gilliam	North-eastern Thailand	2004			
*O. tsutsugamushi*	UT395	EF551309	Karp	North-eastern Thailand	2004			
*O. tsutsugamushi*	UT418	EF551310	Karp	North-eastern Thailand	2004			
*O. tsutsugamushi*	FPW1038	EF551288	TA716-like	Western Thailand	2004			
*O. tsutsugamushi*	FPW2016	EF551289	Gilliam	Western Thailand	2004			
*O. tsutsugamushi*	FPW2031	EF551290	Karp	Western Thailand	2004			
*O. tsutsugamushi*	FPW2049	EF551291	Gilliam	Western Thailand	2004			

NS, not specified; HSP, heat shock protein.

Alignments of the *groEL* gene sequences were used for primer design of the *O. tsutsugamushi* specific real-time PCR assay.

a Based on 56-kDa sequence typing.

The buffy coat samples (from 5 mL of full blood containing EDTA) for real-time PCR were collected from patients with scrub typhus, who gave informed consent, at Udon Thani Hospital, north-eastern Thailand, and at Mahosot Hospital, Vientiane, Laos PDR.

The present study was approved by the Ministry of Public Health, Royal Government of Thailand (Thailand), the Faculty of Medical Sciences Ethical Review Committee, the National University of Laos (PDR Laos) and the Oxford Tropical Research Ethics Committee (OXTREC, UK). The *O. tsutsugamushi* isolates cultured in this study (Table 1) were collected from scrub typhus patients (5 ml full blood in EDTA), which gave informed consent, at two sites in Thailand during 2004-2005.

### Conventional PCR

DNA was extracted with the Wizard SV Genomic DNA purification system (Promega, Madison, WI, USA). Amplification of the partial *groEL* gene was performed using PCR with the previously described [13] primers 5′-GTTGAAGTT/AGTTAAAGG-3′ (forward) and 5′-TTTTTCTTTT/ATCATAATC-3′ (reverse), generating a product of 534–546 bp. A PCR reaction mix consisted of 50 ng of template DNA, 20 nmol of each primer, 1 U of *Taq* DNA polymerase, 1.5 mM MgCl_2_ and distilled water in a total volume of 20 μL. Following 30 cycles of amplification (94°C, 30 s; 44°C, 45 s; and 72°C, 45 s) and a 5-min extension at 72°C on a thermocycler (PTC-200; Bio-Rad, Hercules, CA, USA), the PCR products were subjected to electrophoresis in agarose (2%, w/v) gel (Bio-Rad, Hercules, CA, USA). DNA sequencing was performed commercially by Macrogen, Seoul, South Korea, using BigDyeTM terminator cycling conditions on an automated ABI model 3730XL nucleotide sequencer (Applied Biosystems, Foster City, CA, USA). The nucleotide sequences were edited to equal length (459 bp), and alignments were performed using the Clustal W algorithm [17]. The resultant pairwise percentage divergence was calculated using Megalign software (DNASTAR Lasergene v6 package, DNASTAR, Inc., Madison, WI, USA). The derived *O. tsutsugamushi groEL* sequences were uploaded to GenBank (accession numbers EF551288–EF551310; see Table 1). The reference nucleotide sequences were downloaded from GenBank.

### Real-time PCR

On the basis of alignments of sequences determined from conventional PCR products and sequences available from GenBank, a set of specific primers for the generation of a 160-bp amplicon of the *groEL* gene of *O. tsutsugamushi* was designed using PrimerSelect Version 6.1 software (DNAStar, USA); forward primer, 5′-TTGCAACRAATCGTGAAAAG-3′; and reverse primer, 5′-TCTCCGTCTACATCATCAGCA-3′. The PCR reaction mix contained primers at a final concentration of 200 nM each, 2 μL of DNA template, 10 μL of master mix (QuantiMix Easy, Biotools, Madrid, Spain) containing SYBR green, *Taq* polymerase, MgCl_2_ (4 mM), dNTPs and distilled water in a final volume of 20 μL. The PCR reactions were performed and analysed using a Rotor-Gene 3000 (Corbett Research, Mortlake, NSW, Australia) real-time thermocycler, with an initial holding temperature of 95°C for 5 min, followed by 40 cycles at 95°C for 15 s, 54°C for 15 s and 72°C for 20 s, with fluorescence monitoring at the 54°C annealing step on a predetermined SYBR/FAM channel. Melting curve analysis was performed with increments of 1°C/step (72–95°C) to determine the change in peak fluorescence over time (d*F*/d*T*); positive results were confirmed by electrophoresis of the product in an agarose (3%, w/v) gel in TAE buffer and staining with ethidium bromide (BioRad, Hercules, CA, USA).

To determine detection limits of the assay, plasmids containing the amplified regions of *groEL* (*O. tsutsugamushi* UT176 strain) were generated by ligation into pGEM-T Easy Vectors (Promega, USA) and transfered by transformation into *Escherichia coli*, cultured overnight in a shaking incubator at 37°C in Luria Bertani broth and followed by plasmid extraction using the QIAprep Spin Miniprep Kit (Qiagen, Valencia, CA, USA).

**FIG. 1:**
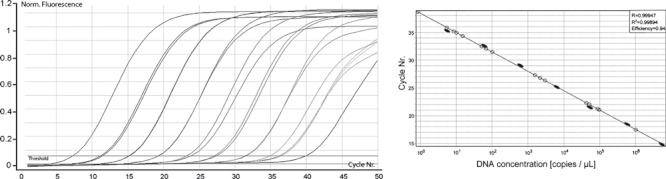
Graph depicting the dilution series (left diagram) of plasmids as external controls to create a standard curve (right diagram) for quantitative analysis. Plasmids are depicted as black ovals, and test samples as hollow circles, the top left circle representing the non-template controls (NTC). Results are per 2 μL of sample, with a limit of detection being 3 copies/μL for both plasmids and buffy coat samples. The copy numbers and corresponding reaction efficiencies were calculated using Rotor-Gene software Version 6.0 (Corbett Research, Australia).

The plasmids were purified and linearized by restriction enzyme digestion with pST1 (Promega, Madison, WI, USA). Linearized DNA was quantified using the Quant-iT PICO Green dsDNA Assay Kit (Invitrogen, Carlsbad, CA, USA). Ten-fold dilution series were used as external controls, and the theoretical number of plasmid copies and corresponding reaction efficiencies were calculated (Rotor-Gene software, Version 6.0; Corbett Research, Australia). Real-time PCR was performed with duplicates of each serial dilution to create a standard curve (Fig. 1).

The specificity of the real-time PCR was determined in three individual runs by assessment of rickettsial reference strains that were grown in VERO cell cultures. The reference strains included SFG members (*R. conorii*, Malish strain; *Rickettsia rickettsii*, Bitterroot strain; *Rickettsia honei*, RB strain; *Rickettsia australis*, JC strain; *Rickettsia sibirica*, 246 strain; *Rickettsia akari*, Kaplan strain), TG members (*Rickettsia prowazekii*, Breinl strain; *R. typhi*, Wilmington strain), and an ancestral group strain (*Rickettsia bellii*). Members of the orders *Anaplasmataceae*, *Bartonellaceae* and *Coxiellaceae* were used as negative controls (*Anaplasma phagocytophilum*, *Bartonella bacilliformis*, *Bartonella henselae*, *Bartonella vinsonii*, *Ehrlichia chaffeensis* and *Coxiella burnettii*). DNA extracts from additional non-rickettsial bacteria (*Burkholderia pseudomallei*, *E. coli*, *Enterococcus faecalis*, *Klebsiella pneumoniae* and *Salmonella enterica* serovar Typhi) all yielded negative results.

## Results

### *groEL* sequence alignments

**FIG. 2:**
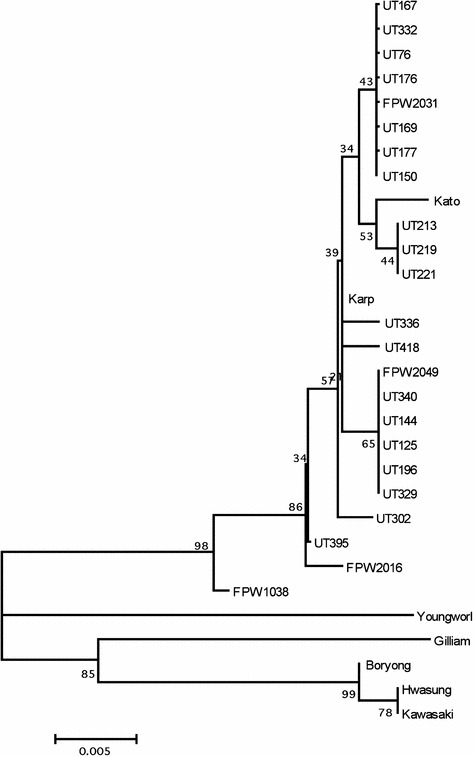
Phylogenetic tree of all 23 new Thai *Orientia tsutsugamushi* isolates, based on *groEL* sequences (459 bp in length), including reference type strains. Owing to the conservative nature of this gene, the discriminatory power is restricted to differentiation among the genera.

The 459-bp *groEL* sequences determined for all Thai isolates were compared with those of reference strains deposited in GenBank, to determine pairwise similarities of nucleotides and amino acids (Table 3; Fig. 2). The overall nucleotide identity for all available *O. tsutsugamushi* isolates, Thai and non-Thai strains, was 98.8% (range: 95.0–100%). The non-Thai *O. tsutsugamushi* reference strains (Karp, Kato, Gilliam, Boryong, Hwasung, Youngworl and Kawasaki) demonstrated a mean intragroup similarity of 96.3% (range: 95.0–100%). The mean nucleotide intragroup identity for Thai *O. tsutsugamushi* isolates was 99.5% (range: 98.9–100%) and the intra-subgroup identities were 99.7% for Karp and 99.6% for Gilliam group members respectively (Table 2, subgroup data not shown).

**TABLE 2:** Mean percentage intragroup identity of all currently available *groEL* gene sequences within the scrub typhus group (STG), typhus group (TG) and spotted fever group (SFG)

Percentage intragroup identity (range)		

Antigenic group	Nucleotides (range)	Amino acids (range)
STG, Thai	99.5 (98.9–100.0)	98.6 (96.7–100.0)
STG, non-Thai^a^	96.3 (95.0–100.0)	89.9 (85.6–100.0)
STG, all available groups	98.8 (95.0–100.0)	96.7 (85.6–100.0)
TG	96.7	91.5
SFG (including *Rickettsia bellii*)	92.8 (86.5–99.8)	81.1 (66.7–99.3)
TG and SFG	92.5 (86.3–99.8)	82.3 (65.4–99.3)
Overall STG, TG and SFG	91.8 (64.0–100.0)	82.2 (26.8–100.0)

a Reported by Lee *et al.*[13].

**Table 3:** Mean intergroup percentage identities of *groEL* nucleotide and GroEL amino acid sequences for scrub typhus group (STG), typhus group (TG) and spotted fever group (SFG) isolates

	Identity (%)						
	
Antigenic group	Isolates (*n* = 39)	STG, Thai	STG, non-Thai	STG, all isolates	TG	SFG	TG and SFG
STG, Thai	23	–	96.7	98.8	67.5	65.6	66.0
STG, non-Thai^a^	7	90.8	–	96.3	67.2	65.7	66.0
STG, all isolates	30	94.5	90.8	–	67.5	65.6	66.0
TG	2	28.6	29.3	28.7	–	91.4	93.2
SFG	7	29.2	29.9	29.3	77.0	–	92.4
TG and SFG	9	29.1	29.8	29.2	81.8	81.1	–

The values depicted in the upper right section above the dividing diagonal represent nucleotide identities; those in the lower left section under the diagonal represent amino acid identities.

a Reported by Lee *et al.* [13].

Comparison of percentage identities of *groEL* gene sequences among antigenic groups showed that Thai strains and non-Thai strains shared a mean nucleotide identity of 96.7%. When Thai *O. tsutsugamushi* strains were compared with TG and SFG strains, the percentage identity levels were found to be 67.5% and 65.6%, with similar values for non-Thai *Orientia* strains when compared to TG and SFG strains, i.e. 67.2% and 65.7%, respectively (Table 3).

### Deduced *groEL* amino acid sequences

The Thai *O. tsutsugamushi* isolates also demonstrated a high overall mean intragroup identity of 98.6% in the amino acid sequences as compared to 89.9% for the non-Thai *O. tsutsugamushi* isolates (Table 2). Further subgroup analysis was based on the full open reading frame (ORF) sequences of the more variable 56-kD outer membrane protein derived from 23 isolates [18] and demonstrated a dominance of the Karp subtype in Thailand (15 of the total 23 isolates) with 99.7% average amino acid identity followed by the Gilliam subgroup (7/23) with 99.6% and the TA716-like group (1/23) with 98.9% amino acid identity (data not shown)

Comparison of deduced GroEL sequences of STG with those of TG and SFG isolates revealed low amino acid identity values of 28.6% and 29.2%, respectively (Table 3).

### Real-time PCR

The assay repeatedly demonstrated a detection limit of < 3 copies/μL of *O. tsutsugamushi*, using serial dilutions of linearized plasmids (Fig. 1). Amplicons with appropriate melting temperatures (average, 84.6°C; range, 84.3–85.1°C) were produced from all *O. tsutsugamushi* plasmids, isolates and clinical samples. All templates derived from clinical isolates and strains belonging to the TG and SFG repeatedly and reliably led to negative results.

Quantitative data obtained with the clinical buffy coat samples, including two samples from Laotian patients, demonstrated copy numbers ranging from 2 to 31 668 copies/μL, with a median value of 64 copies/μL of buffy coat. These values represent bacterial loads in admission samples, corresponding to a median "days of fever" time of 6 (5 – 10) (interquartile range). One sample (UT530) was a clear outlier, both for buffy coat and isolation quantitation, as it demonstrated high bacterial loads in both samples; 28 237/μL of buffy coat and 1 059 061/μL of VERO cell culture at 100% infection of cells as determined by IFA. In cell culture samples, copy numbers ranged between 5.3 × 10^3^ and 1.4 × 10^6^ copies/μL (median 7.0 × 10^4^) of DNA extract (Table 4).

**Table 4:** Description and quantitation data (DNA copy numbers) from characterized clinical buffy coat samples based on the *groEL* real-time PCR assay

		*GroEL* real-time PCR(*T*_m_)		Quantitative real-time PCR (*groEL* copies present in 1 μL of buffy coat or tissue culture pellet)								16S rRNA results	
				
Sample code	Days of fever	TC	BC	1. TC	2. TC	3. TC	Average	1. BC	2. BC	3. BC	Average	% identity	BLAST
UT512	6	84.5	85	655 092	252 376	321 738	409 735	34	90	44	56	99	OT Karp
UT528	6	84.7	84.5	81 500	56 200	49 642	62 447	30	6	6	14	100	OT Karp
UT530	3	84.3	84.4	966 132	861 052	1350 000	1059 061	31 668	23 009	30 034	28 237	99	OT Kawasaki
UT559	6	84.8	84.8	44 272	46 808	74 341	55 140	68	72	28	56	100	OT Karp
UT601	10	84.8	85.1	73 152	70 067	69 893	71 037	582	656	936	725	99	OT Karp
TM1055	5	84.7	84.7	37 416	39 467	33 126	36 670	4	3	4	4	99	OT Kawasaki
TM1084	10	84.5	84.5	9309	7936	5319	7521	23	64	91	59	99	OT Kawasaki

*T*_m_, melting temperature of amplicon; TC, tissue culture; BC, buffy coat; OT, *Orientia tsutsugamushi*; UT, Udon Thani Hospital; TM, Mahosot Hospital, Vientiane, Laos PDR.

## Discussion

The results presented here demonstrate a high level of conservation among the *groEL* nucleotide and corresponding amino acid sequences of contemporary Thai and non-Thai reference isolates of *O. tsutsugamushi.* The high mean nucleotide intragroup identities among Thai *O. tsutsugamushi* isolates can be attributed to the similarities between the two main subgroups, Karp and Gilliam. The *groEL* gene is highly conserved but sufficiently variable to form the basis for genetic target design allowing differentiation of the genera *Orientia* and *Rickettsia*, as sequence analysis demonstrated 99.5% identity within the current 23 Thai isolates, and 98.8% identity for all available *groEL* sequences, including those deposited in GenBank. By comparison, a gene analysis based on sequences encoding the 56-kDa outer membrane protein, covering the full ORF of approximately 1600 bp, demonstrated only 80% identity within the same 23 isolates, underlining the high variability and limitations of this gene as a target for molecular diagnostic assays [18].

At present, only limited DNA sequence data covering the full ORF of the gene encoding the 47-kDa transmembrane protein of *O. tsutsugamushi* are available, but these data and preliminary sequencing results of strains accross Asia (data not shown) are indicative of a high level of conservation.

Currently, three real-time assays are available for the detection of *O. tsutsugamushi*, targeting 16S rRNA genes [19], genes encoding the 47-kDa transmembrane protein [7] and the 56-kDa outer membrane protein [20].

To date, only the real-time PCR assay based on the 16S rRNA gene has been evaluated with a large number of clinical samples, and it has demonstrated a diagnostic sensitivity of 45%, using full blood samples drawn upon admission and IFA as a reference standard.

A recently described nested PCR assay showed an increased sensitivity of 82% [21], using buffy coat as a sample specimen. Both assays had excellent specificities and used reference standard IFA as a comparator, but with different cut-off levels for positivity. As *O. tsutsugamushi* is an obligate intracellular organism disseminating within white blood cells, this assay was based on buffy coat specimens, with the expectation of a concentration effect with higher bacterial loads. A prospective evaluation of the *groEL*-based real-time PCR assay presented here is underway.

On the basis of isolation and 16S rRNA gene sequencing, Karp and Kawasaki strains were found in the samples examined, and no Kato strains were included. Manosroi *et al.* [8] have previously described Karp and Kato strains in the same region, although this was based on a nested PCR assay with use of strain-specific nested primer sets. It is possible that these primers designed for the hypervariable 56-kDa protein-encoding gene could be less specific in detecting Gilliam strains in Thailand. In a recent study, the current group analysed the same 23 isolates by full open reading frame sequencing of the highly specific 56-kDa protein, and revealed the predominance of Karp strains and a substantial presence of Gilliam strains [18]. This new information adds to the existing *groEL* characterization data of five non-Thai isolates [13], suggesting that it is an ideal target for the development of molecular diagnostic assays for *O. tsutsugamushi*.

Stover *et al.* [22] first described the high degree of homology between the (formerly known) *Rickettsia tsutsugamushi* proteins Stp11 and Sta58 and the *E. coli* proteins GroES and GroEL, respectively, and the family of primordial heat shock proteins designated Hsp10 and Hsp60. Although the sequence homology between the Sta58 antigen and the Hsp60 protein family was striking, the antigenic distinction among other bacterial Hsp60 homologues highlighted the uniqueness of this target, suggesting that it may be both a potentially protective antigen and a useful diagnostic reagent for scrub typhus. Park *et al.* [14,15] from Korea took further advantage of these features and incorporated the genetic information for identification, differentiation and characterization within the *Rickettsiae* and *Anaplasmatacae*. The resulting real-time PCR assay proved to be highly sensitive and specific for all tested isolates of *O. tsutsugamushi*. As scrub typhus is endemic in many resource-poor developing countries, *groEL* is a suitable candidate for the application of molecular methods in settings where the costs of establishing a real-time thermocycler are prohibitively high, but alternative methods, e.g. loop-amplified isothermal PCR, an accurate and relatively inexpensive technique, could be used [23,24]. In addition, the reliable and accurate quantitation of bacterial loads allows further investigation of other diagnostic methods and may lead to an improved understanding of the pathophysiology of this important neglected disease. Validation and evaluation in clinical settings in the field are underway.
